# Plant Secondary Metabolites against Skin Photodamage: Mexican Plants, a Potential Source of UV-Radiation Protectant Molecules

**DOI:** 10.3390/plants11020220

**Published:** 2022-01-15

**Authors:** Ana Mariel Torres-Contreras, Antoni Garcia-Baeza, Heriberto Rafael Vidal-Limon, Isaias Balderas-Renteria, Mónica A. Ramírez-Cabrera, Karla Ramirez-Estrada

**Affiliations:** 1 Laboratory of Cell Metabolism, Faculty of Chemistry, Autonomous University of Nuevo León, Pedro de Alba s/n, Ciudad Universitaria, San Nicolás de los Garza 66451, Mexico; ana.torrescns@uanl.edu.mx (A.M.T.-C.); antoni.garciaae@uanl.edu.mx (A.G.-B.); isaias.balderasrn@uanl.edu.mx (I.B.-R.); 2 Centro de Biotecnología FEMSA, Instituto Tecnológico de Monterrey, Avenida Junco de la Vega, Col. Tecnológico, Montrerrey 65849, Mexico; bioraf@gmail.com; 3 Laboratorio de Farmacología Molecular y Modelos Biológicos, División de Estudios de Posgrado, Facultad de Ciencias Químicas, Universidad Autónoma de Nuevo León, Av. Guerrero s/n, Col. Treviño, Monterrey 64570, Mexico; monica.ramirezcbr@uanl.edu.mx

**Keywords:** UV protection, plant secondary metabolites, UVR-damage, antioxidant activity, Mexican plants

## Abstract

Human skin works as a barrier against the adverse effects of environmental agents, including ultraviolet radiation (UVR). Exposure to UVR is associated with a variety of harmful effects on the skin, and it is one of the most common health concerns. Solar UVR constitutes the major etiological factor in the development of cutaneous malignancy. However, more than 90% of skin cancer cases could be avoided with appropriate preventive measures such as regular sunscreen use. Plants, constantly irradiated by sunlight, are able to synthesize specialized molecules to fight against UVR damage. Phenolic compounds, alkaloids and carotenoids constitute the major plant secondary metabolism compounds with relevant UVR protection activities. Hence, plants are an important source of molecules used to avoid UVR damage, reduce photoaging and prevent skin cancers and related illnesses. Due to its significance, we reviewed the main plant secondary metabolites related to UVR protection and its reported mechanisms. In addition, we summarized the research in Mexican plants related to UV protection. We presented the most studied Mexican plants and the photoprotective molecules found in them. Additionally, we analyzed the studies conducted to elucidate the mechanism of photoprotection of those molecules and their potential use as ingredients in sunscreen formulas.

## 1. Introduction

The skin is the largest organ of the body. It acts as an effective barrier against the harmful effects of environmental agents, such as ultraviolet radiation (UVR) and pollution [1].

Exposure to UVR has been associated with a variety of harmful effects on the skin, such as edema, erythema, photoaging, immunosuppression and chronic inflammatory diseases [1,2,3]. UVR is also the major etiological factor in the development of cutaneous malignancies, both melanoma and non-melanoma skin cancers. While melanoma is the less common form of skin cancer (only about 1% of all cases), it causes the most deaths of all skin cancers [4,5].

In the last decades, a 10% reduction in the ozone layer has led to a 20% increase in UVR reaching the surface of the earth. This has led to a significant increase in the incidence of UV-induced human diseases [6,7]. In the United States alone, skin cancers account for nearly 15,000 deaths, 5 million people in treatment, and more than 8 billion dollars in medical costs every year. It is clear that skin cancers represent a major public health concern [8,9].

Although the incidence of skin cancer is increasing, this type of cancer is considered one of the most preventable. Recently it was estimated that more than 90% of skin cancer cases could be avoided with appropriate preventive measures such as regular use of sunscreen. A randomized trial reported that the regular daily use of an SPF 15 or higher reduces the risk of developing melanoma by 50 percent [10].

Plants are constantly irradiated by the sunlight. Because of this, they have developed numerous mechanisms to fight against UVR damage. One of these mechanisms is the synthesis of secondary metabolites with UVR protection activity and antioxidant properties [11,12,13]. These molecules make plants an important source of compounds to avoid UVR damage, reduce photoaging and prevent skin cancers and related illnesses [12,13,14,15].

The aim of this review is to present the principal plant secondary metabolites related to UVR protection and its reported mechanisms. Moreover, to summarize the latest research in Mexican plants related to their UV protection capacity and their potential use in sunscreen formulas. Due to the diversity of climates, Mexican plants represent an important source of UV protectant molecules. Mexican plants synthesize specific secondary metabolites to protect themselves from UVR.

## 2. UV-Radiation Skin Damage

UVR (100 to 400 nm) is classified according to its wavelength in UVA (315 to 400 nm), UVB (280 to 315 nm) and UVC (100 to 280 nm). Approximately 100% of UVC and about 90% of UVB is blocked by the ozone layer, in such a way that UVA represents more than 90% of total UVR daily exposure. UVR classification, characteristics and harmful effects are resumed in Table 1.

UVA radiation is responsible for immediate skin tanning or darkening, premature aging and the suppression of immunologic function [16,17,18]. This type of radiation penetrates deeply into the skin, directly damages DNA and indirectly harms other biomolecules (i.e., nucleic acids, proteins, and membrane lipids) through the production of reactive oxygen species (ROS) [18,19,20]. ROS can potentially lead to oxidative damage of DNA, lipid peroxidation and to the cross-linking of proteins such as collagen. Furthermore, UVA initiates a cascade of inflammatory cytokines via the activation of protein-1 (AP-1) and NF-kB pathways. These last transcription factors upregulate the biosynthesis of matrix metalloproteinase (MMPs). The MMP enzymes degrade elastin and collagen, resulting in skin elasticity reduction and increased wrinkling [18,20,21].

**Figure 1:**
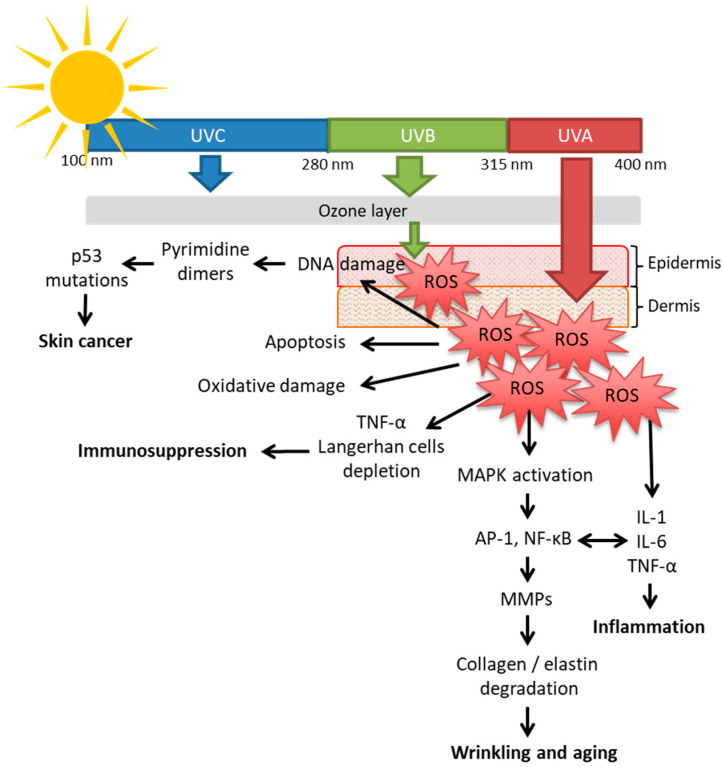
Solar ultraviolet radiation (UVR) and the mechanisms of its main biological effects on the skin. UVC (100–280 nm) cannot penetrate the ozone layer; 2–5% of UVB (280–315 nm) crosses the ozone layer and penetrates the epidermis causing DNA damage and an increase in oxidative stress via ROS formation [1,17]. Approximately 95–98% of UVA (315–400 nm) reaches the skin and penetrates deeper into the epidermis and dermis. This causes diverse harmful effects on the skin, mainly through ROS. (1) Skin inflammation via induction of tumor necrosis factor alfa (TNF-α) and activation of pro-inflammatory cytokines as interleukin-1 (IL-1) and interleukin-6 (IL-6). (2) Skin aging by degradation of collagen and elastin through increasing matrix metalloproteinases (MMPs) induced by the nuclear factor-kB (NF-κB) and the activation protein-1 (AP-1). These last two proteins are also transcription factors for pro-inflammatory cytokines. Cytokines can also amplify AP-1 and NF-kB pathways, enhancing the response to UV radiation. (3) Immunosuppression via TNF-α and depletion of Langerhans cells. (4) Oxidative damage and apoptosis (5) Skin cancer by pyrimidine dimers formation [1,18,20]. All these harmful effects can be prevented by phytochemicals with photoprotection activity.

UVB radiation acts mainly on the epidermal basal layer of the skin and is 1000 times more capable of causing sunburns and harmful skin effects than UVA [22]. The reactions induced by UVB radiation are immediate and include the release of inflammatory mediators (e.g., histamine, serotonin, and prostaglandins) that lead to the development of edema and erythema. In addition, UVB radiation activates numerous signaling pathways that increase the production and activity MMPs, decrease collagen production, induce the accumulation of senescent cells and increase apoptotic cell death and the defective degradation of elastic fibers. Moreover, it has been reported that UVB radiation causes a depletion of the cutaneous defense system and skin cancer [1,22,23]. Figure 1 visually demonstrate the different mechanisms in which UVR causes skin damage.

## 3. Plant-Derived Molecules as Skin Photoprotection Agents

Photoprotection can be defined as a set of measures intended to reduce exposure to sunlight. It represents the main strategy against UVR-related skin diseases. These measures include photoprotective agents in topical sunscreens and oral photoprotectors. The topic photoprotective capacity is determined by the ability of the molecules to absorb, reflect or scatter solar radiation. Oral photoprotectors do not directly protect the skin, but they are able to boost or activate skin mechanisms of photoprotection. New alternatives such as natural photoprotective agents and skin repair stimulators are still being studied [16,24,25,26,27].

In the last few years, botanical ingredients (plant extracts, herbal drugs, plant natural products or isolated plant compounds) have gained the attention of researchers, the cosmetic industry and consumers around the world. These kinds of photoprotective agents also contain large amounts of antioxidant compounds to protect the skin against the harmful effects of UVR [28].

The market for natural cosmetic products is one of the fastest growing in the world. It is expected to reach a value of about 22 billion USD by 2024 [29]. Within the natural beauty product market, the skin care segment holds the highest place. Skincare research and the pharmacological characterization of phytomolecules should evolve together with the demand of the market [30]. It is also important to emphasize that botanical ingredients can be used for dermatologic purposes both as oral dietary supplements as well as in topical cosmetic formulations [31,32].

Recent reports have established the beneficial effects of plant-derived antioxidant compounds in sunscreens since they protect skin against sun damage [33,34,35]. Compounds with aromatic rings, especially phenolic compounds and flavonoids, have a great capacity to absorb both UVA and UVB rays [36,37]. In addition, other families of secondary metabolites, such as carotenoids and alkaloids, have demonstrated photoprotective activity and have been suggested for the development of skin protection products [38,39].

### 3.1. Phenolic Compounds

Phenolic compounds (PCs) constitute one of the most numerous and widely distributed groups of plant secondary metabolites. PCs are divided into three groups: phenolic acids, flavonoids and high molecular weight polyphenols. Nowadays, there are more than 8000 phenolic structures reported [36,40].

The skin health-promoting effects of PCs include photoprotection, anti-inflammatory, antiaging and photo-chemoprevention [41]. The presence of these properties is due to the structure of the PCs. The phenolic rings and hydroxyl groups produce a potent free radical scavenging and antioxidant activity [42].

The PCs antioxidant activity can be explained by different mechanisms of action: (i) inhibition of ROS biosynthesis; (ii) ROS trapping; (iii) reduce metal ions catalysts of ROS synthesis [42,43,44]. The PCs anti-inflammatory mechanisms are not fully elucidated, but it has been reported that phenolic compounds exert this effect by: (i) neutralization of free radicals as ROS and reactive nitrogen species (RNS); (ii) inhibition of activated immune cells, lipid peroxidation and pro-inflammatory mediators, such as interleukin 6 (IL-6) and prostaglandin-E2 (PGE2); (iii) modulation of transcriptional factors, such as nuclear factor-kB (NF- kB) or Nrf-2, in inflammatory and antioxidant pathways; (iv) modification of eicosanoid synthesis [45,46]. The PCs anti-aging effect is associated with the regulation of skin-related gene expressions. PCs upregulate genes involved in oxidative stress protection and skin cell renewal [47]. Additionally, PCs help to maintain the proper skin structure through the induction of elastin and collagen synthesis, inhibition of MMPs via AP-1 and NF-κB activation and the inhibition of collagenases and elastases [36,48]. Furthermore, it has been reported that some plant PCs improve skin conditions by activating DNA mutation repair mechanisms on damaged cells [49].

Non-flavonoid PCs reported to have skin photoprotective activity are: vanillic acid, *p*-coumaric acid, caffeic acid, ferulic acid, rosmarinic acid, chlorogenic acid, gallic acid, tannic acid, as well as resveratrol and curcumin [37,50,51]. Caffeic and ferulic acid are the most studied PCs in terms of photoprotection. These two PCs have been demonstrated to protect phospholipidic membranes from UVR-induced peroxidation by the inhibition of the lipid peroxidative chain reaction [52,53]. Both compounds provided photoprotective effects against oxidative stress, MMP-1 induction and ROS formation in human keratinocytes by the regulation of antioxidant defenses including glutathione (GSH), catalase and glutathione peroxidase (GPx) [54]. Recently it was reported that these two important PCs could protect against UVA-induced melanogenesis through an indirect regulatory effect on the Nrf2 (nuclear factor erythroid 2-like 2) pathway in melanoma cells [55].

Flavonoids are one of the most important classes of phenolic compounds regarding photoprotection. These molecules absorb UVR and reduce ROS oxidative damage. The double bonds present in the flavonoid structure give them a high capacity to absorb UVR, and the hydroxyl groups attached to aromatic rings contribute to their ROS scavenging capacity [37,55]. Currently, there are more than 5000 flavonoids that have been identified and are distributed in different plant species [56]. Flavonoids present protective effects against UVR damage by three main mechanisms: (i) UVR absorption; (ii) direct and indirect (induction of cytoprotective proteins) antioxidant properties; (iii) the modulation of several signaling pathways [56,57]. In addition, flavonoids help to reduce MMPs activity in skin cells via two mechanisms: (i) direct MMPs inhibition and (ii) induction of expression of the tissue inhibitor of MMPs [37].

Epigallocatechin-3-gallate (EGCG), from green tea, is one of the most extensively studied flavonoids in terms of UVR skin photoprotection. EGCG is a potent antioxidant agent that can scavenge ROS. These ROS include superoxide, hydroxyl and lipid-free radicals as well as non-radicals, hydrogen peroxide and singlet oxygen [57]. Moreover, it has been suggested that EGCG protects against UVR induced inflammation, wrinkling and aging via NF-κB and MAPK pathway modulation. This compound has been shown to directly inhibit MMP-2, MMP-9 and neutrophil elastases [58,59]. EGCG has been reported as a photo-carcinogenesis protector. It effectively inhibits tumor incidence, tumor multiplicity and tumor growth in a UVB-treated SKH-1 hairless mouse model when administered either orally or topically [60].

### 3.2. Carotenoids

Carotenoids are tetraterpenoids with a central carbon chain of alternating single and double bonds carrying different cyclic or acyclic end groups [61]. Their extended system of conjugated double bonds gives them photoprotective properties, including the ability to absorb UVR, useful antioxidant capacity by physical quenching of radicals, such as peroxide and singlet molecular oxygen generated during photooxidation, and the inhibition of lipid peroxidation [62]. Furthermore, carotenoids can induce cellular protective responses since they are able to induce phase 2 cytoprotective genes [63]. The mechanisms of photoprotection by dietary carotenoids are reviewed by Stahl and Sies [64].

Lycopene is one of the most studied carotenoids and has been suggested as the most effective against the singlet oxygen radical, which is the most dangerous ROS generated in the skin after sunlight exposure [65,66,67]. Topical application of lycopene protects against UVB photodamage by the inhibition of UVB-induced ornithine decarboxylase (ODC) and myeloperoxidase activities. In addition, it prevents inflammatory responses as well as the cleavage of caspase-3 in the apoptotic pathway [66]. A topical formula based on tomato extract (lycopene-rich) was successfully proven as a form of sunscreen lotion [68].

Additionally, lycopene exerts a photoprotective effect by oral administration. A daily intake of 16 mg lycopene for 10 weeks led to a 40% reduction in skin erythema induced by exposure to solar radiation [65]. Furthermore, it was reported that a diet supplemented with β-carotene (24 mg/day) or a carotenoid mixture consisting of β- carotene, lutein and lycopene (8 mg each/day) for 12 weeks produced protection from UV-induced erythema [66].

### 3.3. Alkaloids

Alkaloids are a group of compounds characterized by the presence of a nitrogen atom in a heterocyclic ring [69]. The photoprotective and antioxidant properties of caffeine, theophylline and theobromine have been studied [39,70]. The most extensively investigated alkaloid in terms of photoprotection is caffeine. The topical and oral administration of caffeine have demonstrated anticancer effects. Topically administered caffeine reduced skin carcinogenesis in UVR-irradiated mice [71]. Oral administration of caffeine led to a decrease in tumor incidence, multiplicity and volume. Moreover, it selectively increased apoptosis in UVB-induced skin tumors in mice [72]. Epidemiological studies indicate that increased caffeine intake is associated with a reduced risk of skin cancer, particularly BCC (basal cell carcinoma) [73,74,75]. The mechanism of this effect involves the increment in apoptosis in cells with defective DNA but, interestingly, not in the normal epidermis cells [76,77].

Recently, the efficacy and safety of some topical sunscreen formulations containing caffeine was addressed. The in vitro functional characterization showed higher SPF values for the caffeine formulation. The in vivo studies also confirmed the higher SPF value. Caffeine acted in synergy (as photoprotector and photostabilizer) together with traditional UV filters such as ethylhexyl methoxycinnamate, avobenzone and titanium dioxide. Thus, caffeine has been shown to be an effective and interesting bioactive compound for use in the formulation of sunscreens [78].

**Figure 2:**
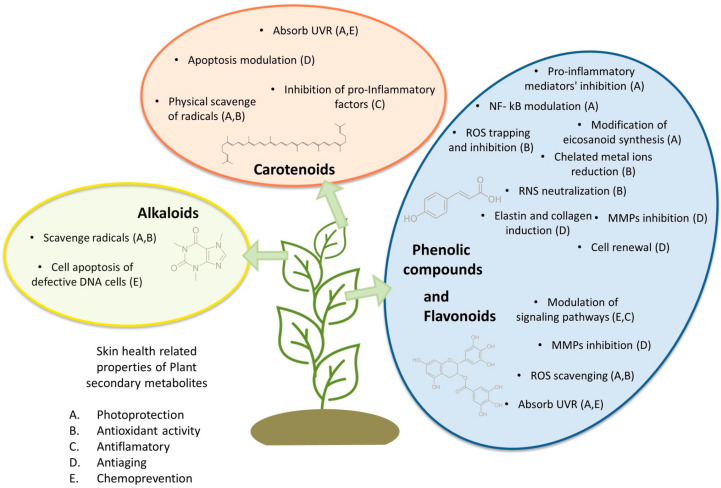
Plant secondary metabolites against skin photodamage. Mechanisms of action and skin-related bioactivities. Matrix metalloproteinases (MMPs) activation protein-1 (AP-1); Nuclear factor-kB (NF-κB), ultraviolet radiation (UVR); reactive oxygen species (ROS), reactive nitrogen species (RNS).

Plants are able to synthesize specialized metabolites to protect themselves from UVR damage. Therefore, these organisms are a unique source of compounds with important photoprotective activities. As we have already reviewed, plant secondary metabolites present several skin health-related properties; these and their related mechanisms of action in the skin are resumed in Figure 2.

## 4. Mexican Plants

Mexico is known as one of the countries with the most plant biodiversity. Due to the different kinds of ecosystems, Mexican plants have developed different strategies to avoid sun exposure damage. Many extracts and molecules isolated from Mexican plants demonstrate protective action against UV radiation (UVR) damage. These are shown in Table 2. Many Mexican plants present specific compounds with important antioxidant and UVR-damage protection effects; this makes them an important source of photoprotective agents. These extracts or molecules can potentially be used in the development of new generation sunscreens.

Different studies have been carried out looking for photoprotective capacity in the extracts of Mexican plants, the elucidation of their secondary metabolites and mechanisms of action. In this section, we summarized the studies conducted on Mexican plants extracts, as well as the molecules in them associated with their UVR protection activities and related mechanisms.

### 4.1. Buddleja Scordioides Kunth and Buddleja Cordata Kunth

More than 120 species comprise the *Buddleja* genus, and they are distributed throughout the world. México harbors 20% of the diversity of this genus in the American continent. [79,80,81]. *B. scordioides* and *B. cordata* are the species most studied due to their UVR protective activities. *B. scordioides* is a shrub occurring in the Chihuahuan desert in Mexico; its extracts have been used as sunscreen in Mexican folk medicine [82]. The photoprotective activity of *B. scordioides* methanolic extract was evaluated as well as two glycoside-like molecules isolated from this plant; linarin (flavonoid glycoside) and verbascoside (phenylethanoid glycoside). Verbascoside presented outstanding photoprotection activity. It exhibited the highest value of SPF (24), measured by guinea pigs bioassays. It also has antioxidant and wound healing properties [83]. Moreover, *B. cordata* bark is used by indigenous people of northern Mexico for skin ailments and inflammations. As well as *B. scordioides*, the phytochemical profile of *B. cordata* methanolic extract revealed high concentrations of verbascoside and linarin (98.75 mg/g and 36.45 mg/g, respectively) compared to other plants. *B. cordata* also contained two other bioactive secondary metabolites, hydroxycinnamic acids (caffeic, ferulic, p-coumaric, and sinapic acids) and terpenes (iridoid, saponin, sesquiterpene, and triterpene) [84,85,86,87]. This extract showed a full coverage absorption in the interval of the UVB spectra. Notably, 60 μg/mL of methanolic extract showed similar UVR absorption (Abs ≅ 0.7) as 10 μg/mL of octyl methoxycinnamate (OMC, octinoxate), the most frequently used UV-filter in sunscreens [84]. It was suggested that the UVR protection showed by *B. cordata* was almost exclusively due to verbascoside. Methanolic extract and isolated verbascoside from the extract showed the same protection activity against UVR-derived damage in both acute and chronic UVR exposition in SKH-1 mice [85]. When compared with the most common plant polyphenols (resveratrol, quercetin, rutin and polydatin), verbascoside is the best candidate for topical photoprotection and skin cancer chemoprevention [86]. Besides chemical photo-stability and direct free radical scavenging, it has been shown to effectively interfere with multiple UVR-sensitive signaling in human epidermal keratinocytes. This led to the inhibition of both inflammatory cytokine expression and cell proliferation, thus producing a great impact on skin cancer chemoprevention. The remarkable photoprotective activity of verbascoside strongly suggests that glycosylation represents the key feature in phenolic compounds that exert an exceptional UVR protection, possibly by giving more stability against the cutaneous chemical barriers [85,87].

Nevertheless, it is well known that the phytochemical profiles of plant extracts can vary depending on diverse biotic and abiotic factors such as soil, latitude, season, time of harvest and others. Therefore, it could be a challenge to standardize phytochemical profiles and ensure the presence of the active ingredient at the indicated concentration [87]. Plant in vitro culture represents an exceptional tool for the production of bioactive phytochemicals as high yields of active compounds can be achieved. Recently the photoprotective activity of *B. cordata* cell culture methanolic extract was reported along with a high concentration of verbascoside (116.36 mg/g dry weight biomass) [86]. Although positive results in photoprotection were obtained, a cytotoxic effect was reported when the fibroblasts (without exposition to UVB) were exposed to either the extract or verbascoside at high concentration (2500 µg/mL and 500 µg/mL, respectively). This cytotoxic effect was suggested to be caused by the previously reported verbascoside capacity to reduce the activity of cytoplasmic protein tyrosine phosphatases implicated in the growth, differentiation and apoptosis of cells [87].

*B. cordata* methanolic extract exhibits a notable antioxidant effect. The antioxidant compounds present reach the intracellular matrix, counteract the ROS damage and stimulate cellular protection. Additionally, oxidative stress reduction was attributed to verbascoside since it has been reported to act as an in vitro DNA protector [86]. The evidence determines the importance of at least two types of bioactive compounds in skin photoprotection: (i) molecules with high capacity to absorb UVR but little absorption into skin cells and (ii) molecules with high antioxidant capacity able to reach the intracellular matrix in the epidermal cells. The first group of molecules directly avoids UVR damage. The second group of molecules decreases the oxidative stress generated indirectly by the UVR through ROS production.

Future research should be conducted to find out if *B. scordioides* and *B. cordata* methanolic extracts can be used in the formulation of sunscreens. Additional in vivo studies regarding the use of these extracts in chronic UVR exposition, as well as the in vivo evaluation of the toxicity, are required.

### 4.2. Echinacea spp.

*Echinacea* is a genus naturally occurring in North America. It comprises nine species throughout Mexico, generally found in the south and the Gulf regions [88]. The interest in this plant has increased due to its promising effects on human health and well-being. *Echinacea* spp. is extensively used in the treatment of certain diseases, mainly as an immunomodulator, and there is scientific evidence regarding the safety of products derived from this plant [89,90]. A patented sunscreen formulation successfully included *Echinacea* extracts, *Cynara scolymus* extract and organic or inorganic synthetic sun filters. This obtained promising results and improved the photoprotective properties of the sunscreen formula by reducing UVR skin damage. These photoprotective properties are related to the already known antioxidant activity of *Echinacea* spp. extracts [91,92,93,94]. The antioxidant effects of this genus are provided by caffeic acid and its derivatives (hydroxycinnamic acids) as well as flavonoid content in the *Echinacea* extracts. However, due to the chemodiversity, the amount among the species differs. The scavenging power of these molecules has the following order, from more active to less active: caftaric acid, chlorogenic acid, caffeic acid, cynarin, echinacoside and cichoric acid [95]. A recent study revealed that cichoric acid provides UVR protection in plants cells by acting as a sunscreen and antioxidant agent [96]. Additionally, some caffeic acid derivatives, such as cichoric acid and chlorogenic acid (from *E. purpurea*)*,* have been functionally linked to anti-inflammatory and wound healing properties when applied topically [97].

Dermatological formulations, cream and gel, containing *E purpurea* extract have been tested. The stability of the extract and formulation as well as the effect on skin irritation, hydration level and wrinkle reduction, were evaluated. The study reported low stability of *E. purpurea* extract, especially regarding the total phenolic content and the antioxidant activity. However, the formulations were shown to improve the hydration of the skin and decrease skin wrinkles without inducing any short-term or long-term irritation [98]. Phenolic compounds as well as the antioxidant activity of the formulations, decreased faster in gel than in cream. Gel formulation is water-based, thus oxygen dissolved in water can cause phenolic compounds to oxidize faster than in cream. Conversely, in a cream formulation, some phenolic compounds could be contained in the oil phase, which avoided oxidation. Formulation techniques that allow more stability to the extract, such as encapsulation, should be further evaluated in order to enhance the phenolic compound stability, antioxidant activity and shelf life of the formulation.

Similar to other members of the Asteraceae family, *Echinacea* contains some polyacetylene compounds that are phototoxic and cytotoxic [99]. Fortunately, it is possible to inactivate these compounds by minimal processing [100].

In 2005 *E. purpurea* was included in the European Medicines Agency official list of herbal substances for use in traditional herbal medicinal products [101]. This shows that these plant genus extracts can indeed be real ingredients for the development of sunscreen and dermatological products. However, more studies are necessary, and a phytochemical profile related to UVR protection is required to determine the photoprotective capacity and mechanisms of the molecules contained in this plant´s extracts.

### 4.3. Galinsoga parviflora Cav. and Galinsoga quadriradiata Ruiz & Pav

*G. parviflora* and *G. quadriradiata* belong to the *Asteraceae* family and are among the most common weeds in Mexico. The chemical composition, photoprotector activity and traditional uses are similar in both species, mainly containing flavonoids and hydroxycinnamic acids [102,103]. Many *G. parviflora* preparations have been applied in traditional medicine around the world, showing dermatological therapeutic activities for eczemas and lichens [103].

Aqueous extracts from *G. parviflora* and *G. quadriradiata* showed antioxidant and photoprotective activities. These two extracts decreased apoptosis and ROS production in Normal Human Dermal Fibroblast (NHDF) irradiated with UVA or UVB [102]. A decrease in lactate dehydrogenase (LDH) release, a decrease of apoptotic cells and the inhibition of intracellular ROS production were observed in UVA exposed NHDF cells, pretreated with two new isolated caffeic acid derivatives (2,3,5(2,4,5)-tricaffeoylaltraric and 2,4(3,5)-dicaffeoylglucaric acid) from *G. parviflora* in a concentration-dependent manner. Additionally, an increase was observed in cell viability and glutathione (GSH) production. Moreover, both compounds were able to increase heme oxygenase (HO-1) expression, an enzyme that is part of the antioxidant defense in human skin cells. They also activated Nrf2 (nuclear factor erythroid 2-like 2) transcription factor in the tested fibroblast cell line in order to maintain the cellular redox status and to protect against oxidative damage [103].

The UVA photoprotection of the caffeic acid derivatives isolated from *G. parviflora* is due to the enhancement of the intracellular antioxidative enzymes through the induction of transcription factors of antioxidant genes. The polyphenolic compounds present in this plant extract have different mechanisms of action in oxidative stress suppression against UVR-caused damage. A mixture of different plant secondary metabolites exerting a variety of mechanisms of action could be the strategy to protect the skin broadly against UVR. Therefore, *G. parviflora* and *G. quadriradiata* plants and their extracts present an excellent potential for being used in sunscreen formulations.

### 4.4. Helianthus annuus L.

The wild sunflower is a native plant of northern Mexico [104]. It is a robust perennial that measures 1–3 m in height. Its characteristic flowers are surrounded by yellow external petals and brown internal inflorescence. Sunflower seeds and sprouts contain high levels of flavonoids, phenolic compounds, polyunsaturated fatty acids and vitamins. These compounds give antioxidant, antimicrobial, anti-inflammatory and wound-healing activities to the plant extracts [105].

This plant has been used in folk medicine for skin-related illnesses, and it is still used and studied for the treatment of skin disorders as well as skin care [106,107].

Recently, the *H. annuus* flower ethanolic extract, containing chlorogenic acid and caffeic acid as active and major components, demonstrated a photoprotective activity against UVB damage in UVB irradiated human dermal fibroblasts. The ethanolic extract (50%) inhibited UVB induced MMPs and ROS production, as well as vascular endothelial growth factor (VEGF) and inflammatory cytokines secretion. Furthermore, sunflower extract demonstrates an antiphotoaging action by the activation of Nrf2, upregulation of TGF-β1, promoting procollagen type I synthesis, downregulation of AP-1 and MAPK phosphorylation [108]. This evidence suggests that *H. annuus* flower extract contains promising photoprotective molecules against UVB-induced skin damage.

The formulation of sunscreen is crucial in order to effectively produce UVR protection. Recently it was reported that nanoemulsion formulation is more effective in sunscreen cosmetic use as compared with simple emulsion when sunflower oil is used [109]. The SPF value in all the nanoemulsion preparations tested with *H. annuus* was higher than the simple emulsion since the nanoemulsion preparation has a smaller globule than the emulsion and hence can absorb more UVR. These results suggest that the phytochemicals and their mechanisms of action to exert photoprotection are decisive for the design of an adequate formulation of each extract.

### 4.5. Larrea tridentata (DC.) Coville

As a member of *Zygophyllaceae* family, this particular evergreen shrub is distributed abundantly in the driest regions of northern Mexico, living in arid and very arid ecosystems. The resin secreted by the bark of this plant has shown insecticidal and fungicidal activity. Among a vast number of chemical compounds in *L. tridentata* leaves, nordihydroguaiaretic acid (NDGA) highlights as the most abundant metabolite with the best antioxidant activity [110].

In north-Mexican traditional medicine, this species is widely used to treat many infectious diseases, as well as to remedy some states and illnesses, including aging and inflammatory processes [111].

Several studies have demonstrated that NDGA holds important anticancer and antioxidant activities [112,113]. Additionally, there is evidence suggesting this compound can inhibit UVB induced signaling pathways related to skin carcinogenesis as well as redox signaling associated with UVB irradiation [114,115,116,117]. NDGA significantly inhibited UVB-induced signaling pathways in human keratinocyte cell line HaCaT. This suggests a high potential for the prevention of skin cancer. In vitro assays demonstrated the inhibition of AP-1 activation and c-fos transcription factor. Also, NDGA reduces 40% of PI 3-kinase enzyme activity and prevents phosphorylation of serine/threonine kinase Akt [118].

All of this evidence suggests that NDGA can efficiently prevent UVB induced skin cancer by two mechanisms of action. First, by the inhibition of signaling and protein expression that may be associated with the development of skin carcinogenesis and secondly, by preventing the harmful effects of ROS due to the antioxidant action of this polyphenolic compound. However, more studies are required to evaluate the efficacy and security of *L. tridentata* extracts containing NDGA and other polyphenolic compounds.

### 4.6. Lippia graveolens Kunth

*L. graveolens* is a native Mexican shrub commonly known as “flor de oregano”, and it belongs to the *Verbanaceae* family [119]. The areal part of this plant is traditionally used as a condiment as well as to treat some illnesses, including inflammation and related disorders [120]. The *Lippia* genus has demonstrated antioxidant activity. The polar extracts of three species acted as ROS scavengers, reduced nitric oxide (NO) release and inhibited cyclooxygenase in macrophage cells [121].

The methanolic extract of *L. graveolens* showed a maximum absorption peak in the UVB region (λ = 280 nm), indicating a putative sunscreen effect. This effect was evaluated in an *E. coli* model. The cell death was reached at 6 min without protection, while with the methanolic extract treatment, it took 150 min. Furthermore, the *L. graveolens* methanolic extract demonstrated a photoprotective effect from UV exposure in hairless SHK-1 mice, showing a reduction of lesions close to 100%. Additionally, topical administration of the methanolic extract was reported to have protected against chronic UVB-induced skin cancer in SKH-1 mice by reducing the incidence and multiplicity of tumors. Phytochemical profile analysis revealed the presence of irinoids, three flavanones with a similar structure (apigenin, pinocembrin and naringenin), 23 flavonoids and naphtoquinoid. These molecules showed a high penetration capacity in the skin, suggesting oxidative stress protection to the primary layers of the dermis [122]. Apigenin, pinocembrin and naringenin seem to be the main photoprotective compounds in *L. graveolens*. Pinocembrin and naringenin have a maximum absorbance value within the UVC and UVB range but not in the UVA, while naringenin showed higher absorbances within the UVC zone and pinocembrin in the UVB. Only apigenin absorbed the entire UV spectra. This showed a higher SPF capacity. Therefore, apigenin could be considered as a broad-spectra photoprotector. Additionally, apigenin, pinocembrin and naringenin showed protection in UVB-induced DNA genotoxicity in HEK-293 and *E. coli* cells acting as a UV filter [123].

A sunscreen formula using an extract of a species from the same genus demonstrated in in vitro and in vivo assays UVR protection effectiveness [124]. All this evidence suggests the great potential of *L. graveloens* as a UV filter in dermatological sunscreen formulations. Further studies with this Mexican plant are required to utilize the full potential of its extracts in sunscreen formulae.

### 4.7. Malpighia glabra L.

*The Malpighia* genre comprises 12 endemic Mexican species of shrubs and small trees belonging to the *Malpighiaceae* family. It grows naturally in the Yucatan peninsula and southern states of Mexico, extending to Central America [125]. The fruit juice from *M. glabra* contains high vitamin C content, as well as flavonoids, carotenoids and anthocyanins. All these compounds have many biological activities, including antioxidant effects. Thus, this plant exhibits a large potential for application in the cosmetic industry [126].

An evaluation of the in vitro antioxidant and photoprotective activities of dried *M. glabra* extract in emulsions with chemical filters (widely employed in the cosmetic industry) demonstrated free radical scavenging exclusively in the formulations with the extract. Additionally, formulations combining fruit extract and chemical filters in emulsion proved to be the most stable. In vitro SPF and antioxidant tests showed a synergistic effect on skin protection from UV irradiation damage. Although the extracts by themselves did not present typical sunscreen behavior, the synergistic effect with commercial UV filters and the antioxidant activity is what makes the extracts of this plant of interest. [127]. More studies are necessary to understand the mechanism related to this synergistic behavior and the mechanisms of photoprotective activities in this plant. The phytochemical profile is unique in each plant and is important to the formulation of dermatological sunscreen products. Determining the phytochemical profile of *M. glabra* extract will help to elucidate the mechanisms of action of this plant.

### 4.8. Pipper umbellatum L.

*P. umbellatum*, (synonyms: *Pothomorphe umbellata*, *Lepianthes umbellate*, *Heckeria humbellata*, *Peperomia umbellata*) is a perennial shrub with no clear origin. However, it is distributed throughout Mexico, Central America, South America and West Indian islands [128]. 4-nerolidylcatechol is considered the main secondary metabolite found in *P. umbellatum* and exhibits remarkable antioxidant activity [129]. However, the methanolic extract of *P. umbellatum* showed higher antioxidant activity than isolated 4-nerolidylcatechol. This suggests the important role of the other secondary metabolites present in *P. umbellatum* [130]. Although a specific and complete phytochemical profile of *P. umbellatum* has not yet been reported, the presence of alkaloids, flavonoids and triterpenes has been detected [131].

As we mentioned in the introductory section, antioxidants applied topically can strengthen the endogenous skin UVR damage protection system and represent an important strategy to reduce UVR damage. UVR can penetrate and cause damage to the deeper layers of skin. Therefore, an antioxidant topical product must permeate deeply into the skin to obtain the desired effect. The percutaneous absorption of the root extract of *P. umbellatum* and isolated 4-nerolidylcatechol into a gel and emulsion formulation was studied for further development of a sunscreen product. The results concluded that the four formulations tested present adequate percutaneous penetration, but 4-nerolidylcatechol showed better results in terms of absorption. On the other hand, *P. umbellatum* root extract gel-formulation presented better antioxidant potential due to the additional compounds present in the extract [132]. In order to understand the mechanisms involved in the antioxidant activity of *P. umbellatum* root extract into the skin, the effect of topical administration of this extract was evaluated. In studies on hairless mice, the impact on the antioxidant skin components in UVR induced damage was evaluated. Depletion of alpha-tocopherol, one of the main skin antioxidants, was avoided on hairless mice exposed to UVB when a *P. umbellatum* extract gel was applied to their skin [133]. This work was the first to demonstrate the effectiveness of *P. umbellatum* as a photoprotective agent. In 2005, a reduction in chronic skin damage and photoaging was observed in hairless mice chronically exposed to UVB radiation and previously treated with 0.1% *P. umbellatum* root extract in a carbomer gel as a vehicle [134]. After this finding, the same research group evaluated the photostability and photoprotection potential of this formulation. The *P. umbellatum* root extract gel was stored at 5 and 25 °C for 3 months and was stable throughout this time [135]. In order to understand the mechanism of the antiphotoaging of this plant, a study conducted by Ropke et al. investigated the effect of *P. umbellatum* root extract on matrix metalloproteinases (MMP2 and MMP9). The results obtained were the in vitro and in vivo inhibition of MMP9 activity by the root ethanolic extract [136]. After two years, another mechanism-related study was conducted. In that work, the authors demonstrated the positive effect of the *P. umbellatum* root extract when it was applied to the UVB-radiated skin of hairless mice. Previous application of *P. umbellatum* root extract significantly reduced the number of thymine dimer-positive cells and apoptotic sunburn cells in single UVB exposure hairless mice. Additionally, an increase was observed in the p53 and proliferating cell nuclear antigen-positive cells. During repeated UVB exposure, the *P. umbellatum* root extract was able to inhibit the hyperplasic response and produced an increase in p53-positive cells in the epidermis of hairless mice [137]. Together, these findings suggest the great potential of this plant for the development of UVR protection products. Further studies are required to elucidate the role of phytochemicals and the molecular and biological mechanisms involved in the photoprotection, particularly in cell-cycle arrest and DNA damage repair caused by *P. umbellatum*.

### 4.9. Polypodium leucotomos (L.) J.Sm.

*P. leucotomos* is a Mexican native fern belonging to the *Polypodiaceae* family [138]. This plant has long been used in South America and Spain to treat psoriasis [139]. Moreover, Native Americans have used this plant for anti-inflammatory skin purposes [140].

Phenols and polyphenols are the main photoprotective compounds found in *P. leucotomos*. The most abundant molecules include benzoates, cinnamates, quinic, shikimic, malic, ferulic, coumaric, vanillic and chlorogenic acids [141]. All these molecules possess physical and chemical photoprotective features [142]. The effects against photodamage and dermatologic applications of *P. leucotomos* are well-studied and documented by in vivo and in vitro studies and have been recently reviewed [143,144,145]. We will describe some of these interesting effects below.

Photoprotective mechanisms of *P. leucotomos* include strong antioxidant activity which is attributed to hydroxycinnamic acid family polyphenolic compounds [146]. Inhibition of MMP-1 and reduction of lipid peroxidation induced by UVR was observed in UVA and UVB irradiated fibroblasts and keratinocytes treated with *P. leucotomos*, suggesting a cell membrane protective and antiphotoaging effect [147]. In human keratinocytes, the hydrophilic extract of *P. leucotomos* extract inhibits pro-inflammatory tumor necrosis factor alfa (TNF-α) factor and nitric oxide synthase (iNOS) expression. It also inhibits the transcriptional activation of nuclear factors NF-κB and AP-1, apoptosis and MMPs production induced by sun radiation. This study demonstrates the cytoprotective effect of *P. leucotomos* extract and its multifactorial mechanism. Therefore, the photoprotective effects of this plant extract are not only related to its antioxidant capacity [148].

Topical effects are not the only ones reported for this plant, as oral administration also has proven to be effective against UVR damage. Oral administration of *P. leucotomos* extracts demonstrated photoprotective properties in hairless rat models. The hydrophilic extract of this plant enhanced antioxidant plasma capacity and reduced glutathione oxidation in blood and epidermis. Additionally, treatment with this extract inhibited UVR-mediated Langerhans cells depletion, increased the number of p53^+^ cells and reduced proliferating cells. These studies demonstrated *P. leucotomos* hydrophilic extract is able to reduce systemic UVR damage and regulates the endogenous antioxidant enzymes [149,150]. Therefore, *P. leucotomos* can be used as an oral antioxidant and photoprotective complement to topical sunscreens with outstanding effects.

Clinical studies have been performed on healthy volunteers. *P. leucotomos* extracts showed a notable efficacy in erythema and skin photosensitization reduction in human volunteers exposed to limited UVA radiation. Oral administration was shown to reduce sunburn, decrease the loss of Langerhans cells and diminish mastocytes infiltration in the skin. It inhibited DNA’s damage and mutagenesis by preventing UV-induced accumulation of cyclobutane pyrimidine dimers. The reported photoprotective dose in healthy humans was 7.5 mg/kg [151,152,153].

These insights, together with the corresponding pharmaceutical industry efforts, resulted in the successful production and commercialization of a concentrated hydrophilic extract of *P. leucotomos* leaves [154]. This product constitutes one of a new growing generation of natural photoprotective products that can be used either systemically (oral intake) or topically.

Recently a booster effect of *P. leucotomos* was reported when applied topically [155]. The extract, besides the photoprotection, increased immune protection. It elevated the contact hypersensitivity factor and the human immune protective factor of the sunscreen formulations [155]. These kinds of biological activities in natural products are precisely what is being sought in the new generation of sunscreens, not only active ingredients that filter the UVR, but also products that have additional benefits to the skin as an anti-inflammatory, anti-aging and immune protective biological activity.

### 4.10. Portulaca olearacea L.

*P. oleracea* is a member of the *Portulacaceae* Family, is considered an edible vegetable and herbal medicine. It is commonly known as purslane, verdolaga, pigweed, little hogweed or pusley. It is distributed worldwide in tropical regions, and in some areas, is considered an invasive weed [156]. There is evidence that this plant has been present in North America since approximately the year 1000 B.C. [157]. In addition, in Mexico, archaeological records showed evidence of the ancient use of this plant [158]. Due to its weed-like characteristics, purslane occurs widely throughout the Mexican nation.

Purslane is well-known as a medicinal plant in diverse traditional medicine systems with a wide range of pharmacological activities. It is listed by the World Health Organization as one of the most used medicinal plants and named “the global panacea”. Within its pharmacological activities, it includes some skin-related uses such as pruritis treatment (itchy skin), urticaria, sores, erysipelas, eczema, burn and wound healer and to ameliorate skin allergies [159,160].

Phytochemical screening revealed that this plant possesses a wide range of secondary metabolites, including phenolic acids, flavonoids, alkaloids, terpenoids, steroids, tannins, saponins and organic acids [161,162,163,164].

Flavonoids have been reported as the most active compounds of purslane. Kaempferol, apigenin, luteolin, myricetin and quercetin are its major flavonoids. Portulacanones A-D are homoisoflavonoids (exclusive to purslane) with a unique chemical structure and have been isolated from aerial parts of the plant [163,164].

Modern pharmacological studies revealed skin health-promoting activities, such as antioxidant, anti-inflammatory, anti-wrinkle and anti-aging properties when using purslane extract topically [162,165,166].

Antioxidant activity of *P. oleracea* methanol extract was tested, and the study reported a potent free radical scavenging related to the aging process and skin wrinkling. It also provided some photoprotective action [165].

Recently, it was described that the protective effect of the *P. oleracea* extracts against UVB-induced damage in human epithelial keratinocytes and fibroblasts effectively reduced cell death and apoptotic DNA cleavage after UVB irradiation. The extracts did not reveal cytotoxicity. Therefore, *P. oleracea* extracts can be used safely as an effective cosmetic ingredient to prevent UVB-induced skin damage [156,166]. Further research is needed in order to elucidate the phytocompounds responsible for the reported and specific biological activity and mechanisms of action.

### 4.11. Solanum lycopersicum L.

Tomato is a *Solanaceae* family member native to Mexico. It is widely distributed throughout Mexico and extends all the way to South America [138]. It grows in tropical climates with annual cycles and has been cultivated since the Aztec civilization due to its gastronomic properties and health uses [167]. The tomato plant has a large list of diverse biological activities, including antioxidant, anti-inflammatory, anticarcinogenic and others [168,169,170]. Furthermore, it is considered a protective food because it provides important nutrients such as lycopene, beta-carotene, carotenoids, flavonoids, vitamin C and hydroxycinnamic acid derivatives [171]. The most abundant carotenoid in tomato fruit is lycopene. This isolated molecule and carotenoids enriched tomato extracts were tested as oral photoprotectors against UV-induced erythema in volunteers. The best photoprotective effect was observed in the two groups with tomato extract treatment [172].

Recent research showed that lycopene, concurrent with naringenin, have photoprotector activity against oxidative stress in human dermal fibroblasts exposed to UVA [173]. Moreover, tomato consumption effect in skin cancer development was tested in SKH-1 murine skin after chronic UVB exposure. Mice fed with tomato diets presented a significantly lower tumor number compared to controls. This suggested that lycopene levels and glycoalkaloids derivates in tomatoes can modulate keratinocyte carcinomas development [174].

The evidence shows lycopene and tomato extracts as potential sources of oral photoprotectors. The mechanisms involved are still unknown. Further investigations are needed in order to elucidate the role that tomato phytochemicals play in the mediation of keratinocyte carcinomas as well as in oral photoprotection.

### 4.12. Spondias purpurea L.

Ciruela Mexicana, also known as jocote, or hog plum. *S. purpurea* is a small tree native to Mexican and Central American tropical dry forests [174,175]. Edible parts of different ecotypes of *S. purpurea* present high quantities of total phenolic compounds and a positive correlation with the antioxidant capacity [176,177].

Methanolic extract of the peel was used to evaluate the photoprotector effect in a putative sunscreen. The metabolic profile of this extract was amplified by analysis by HPLC-MS. The 30% *S. purpurea* extract formulation showed excellent UVA and UVB light protection with an SFP value of 43.01 ± 0.81 and a high antioxidant capacity. The phytochemical profile revealed the following secondary metabolites: dicaffeoylglucose, HHPD-galloyl-glucose, galloyl-bis-HHPD-glucose, quercetin-3-*O*-rutinoside (rutin) and quercetin. Quercetin has a higher antioxidant capacity among flavonoids. Rutin is found in the species of the *Spondias* genus and can suppress inflammatory signals derived from UV exposure [178]. However, this secondary metabolite has low solubility in water. Some attempts to enhance its solubility were made by the development of nanoparticles improving the mentioned solubility and the SPF value [179].

Recently, the in vitro photoprotective activity of a stem bark hydroethanolic extract of *S. purpurea* was evaluated [180]. In this extract, 30 different compounds were identified. These compounds included 5 simple phenolic acids, 5 hydrolysable tannins, 11 flavonoids and derivatives, 2 benzophenones and 4 simple acids glycosylated. The SPF value for the extract was from 14.37 (0.2 mg/mL) to 26.16 (2 mg/mL). Six formulations were evaluated. Two of these contained octyl methoxycinnamate as the active ingredient concurrent with *S. purpurea* extract. The four others contained only the extract as the active ingredient in different concentrations. The formulations containing only the *S. purpurea* extract showed an increase in the SPF value as the concentration of the extracts (phenolic compounds) increased. This demonstrates that the photoprotection is due to the phenolic content in the extract [180]. The sunscreen formulations with the same plant extract in combination with the commercial UV filter did not increase the SPF value. This result demonstrates that there was no synergistic action between components of the formulations.

More studies to elucidate the mechanisms of action are required to better understand the effects of this plant extract. The studies presented herein demonstrate that *S. purpurea* is a promising source of photoprotective agents for new sunscreen formulations.

### 4.13. Theobroma cacao L.

*Theobroma cacao* or cocoa is a native Mexican tree. It was considered a divine food by pre-Hispanic Mexican civilizations. Many documents have reported its health benefits and medical uses. Large numbers of its products, extracts and isolated bioactive compounds have demonstrated pharmacological effects [181]. Cocoa is one of the richest foods in flavanols and phenols. Epicatechin and catechin are their most representative flavanols [182]. Different studies have demonstrated the UV photoprotection effect derived from regular consumption of cocoa. The mechanism remains unexplored, but it is assumed that it is mediated by the effects of flavanols in the erythema-inflammatory response [183]. The alkaloid theobromine also presents important photoprotective and antioxidant properties, improving and maintaining skin health [70].

Human clinical trials showed that ready-to-eat cacao products rich in flavanols conferred UV-photoprotection. In this study, a Cocoa beverage enriched in flavanols and one with a low concentration were compared. Subjects fed with the flavanol-rich beverage showed a 25% reduction in the UV-exposure induced erythema [184]. A similar randomized, double-blind clinical study was performed. In this study, subjects were fed with chocolate containing more than 3% of flavanols or chocolate with a low concentration of flavanols. The dietary consumption of chocolate rich in flavanols conferred photoprotection to the UV exposure in comparison to the low flavanols concentration group, which did not [183].

A study using cocoa pods, usually discarded, revealed a high content of total phenols and flavanols. Thus, cocoa pods were processed to evaluate their in vitro anti-wrinkle effect, UV-absorbance and anti-inflammatory capacity. Cocoa pod extract was able to absorb the UVB wavelength range where a commercial sunscreen did not. Moreover, cocoa pod extract exhibited higher antioxidant capacity than pine bark extract as well as stronger inhibition of collagenase and elastase [48]. These insights demonstrate the potential of *T. cacao* to be used as a sunscreen ingredient. It can be administrated orally (beverage) or topically (pods extracts). Studies related to dosage and toxicity are needed to complement the findings in the existing studies.

### 4.14. Yucca periculosa Baker

*Yucca* is an extended genre native to Mexico. It comprises many species, and 40–50 have been used for medical purposes, including skin-related pathologies. North American Indians used the brewed leaves to treat psoriasis, hair loss and sunburns. Nowadays, this plant is used in alternative therapy as herbal medicine in the pharmaceutical form of capsules containing extracts for skin care or even thrombosis prevention [185].

*Y. periculosa* seems to be the most promising species within the genre in terms of photoprotection. This tree grows in arid regions, and it mainly occurs in the states of Oaxaca, Puebla, Tlaxcala and Veracruz in Mexico [186]. Important polyphenolic compounds with antioxidant activity have been found in this plant species. Methanolic extract contains resveratrol, trans-3,3′,5,5′-tetrahydroxy-4′-methoxystilbene and naringenin [187]. *Y. periculosa* methanolic extracts and isolated compounds revealed a high photoprotective effect against UVB skin damage and cell death in guinea pigs and prokaryotic cells, respectively [187]. Resveratrol and methoxystilbene presented the best UV protection characteristics, absorbing in the UVB region (λmax 305 nm and λmax 316 nm, respectively). In addition, they did not show UVB-induced inflammatory changes in the histopathological observations [187]. Resveratrol occurs in both trans- and cis- forms in the plant, and the effect between them may vary. Trans-resveratrol is the most common and stable isoform, while cis-resveratrol is unstable and it is not possible to commercialize it. It was reported that the trans form of resveratrol is transformed into the cis when exposed to UV radiation at wavelengths of 254 nm and 366 nm. This is a point for discussion if a sunscreen-like product containing resveratrol is desired to be developed [188]. However, more studies are needed to better understand the mechanism related to the photoprotection of this plant.

### 4.15. Opuntia spp.

*Opuntia* spp. belongs to the *Cactaceae* family, which is reported to contain around 4000 species. This cactus is native to México and is widely distributed in America, Africa, Asia, Europe and Oceania [189]. Mexico has nearly 675 species, and 84% of them are endemic [190]. Among all of the species, *Opuntia ficus-indica* L. Mill is the most widely distributed. This plant has been used in traditional folk medicine for the treatment of burns, wounds, edema, indigestion, diabetes, hypertension, rheumatic pain, hypercholesterolemia, among other diseases and conditions around the world. The wide variety of beneficial effects on human health attributed to this plant (from both fruits and cladodes) has increased the interest in it, not only from the nutritional side but from the pharmacological industry as well. Many researchers have studied and linked the phytochemical compounds to the health-promoting activities attributed to this plant, and some have even discovered new ones.

Although the beneficial properties of *Opuntia* on the cutis (as favoring cutaneous reparative processes that stimulate cell renewal and collagen production) are historically well known [191], little has been explored regarding photoprotective properties.

Recent research has demonstrated that a simple aqueous extract of *O. ficus-indica* has strong antioxidant properties able to counteract the negative UVA radiation effects induced in irradiated human keratinocytes [192]. Human keratinocytes treated with the extract before being exposed to UVA radiations showed a marked inhibition of stress-induced processes, such as free radical production, lipid peroxidation and GSH depletion. This protective effect was attributed to eucomic and piscidic acids (phenolic compounds) present in the Opuntia aqueous extract [192].

A hydrolyzed *O. humifusa* (Raf.) Raf. aqueous extract was reported to protect from UVB-induced skin degeneration in HaCaT cells and hairless mice after oral or topic administration [193]. This effect was reported to occur by inducing dermal hyaluronic acid production and the prevention of unhealthy skin states such as water loss and erythema formation. This effect is related to the flavanol content, particularly quercitrin and taxifolin [193].

Later research suggested that the photoprotection activity of *O. ficus-indica* is mainly due to the antioxidant property of opuntiol, the major flavonol reported in *Opuntia* extracts [194]. The effect of opuntiol (isolated from *O. ficus-indica*) against UVA-mediated oxidative damage in mouse embryonic fibroblast cell lines (NIH-3T3) was evaluated. Opuntiol was reported to be an excellent antioxidant molecule in different free radical scavenging systems. It was also able to prevent UVA radiation oxidative damage indices such as lipid peroxidation, DNA strand breaks and apoptotic incidence in NIH 3T3 cells [194]. Therefore, *Opuntia* cladodes represent a suitable, low-cost and high value-added substrate for the isolation of phytochemicals to be used in pharmaceutical and/or cosmetic applications for skin care and photoprotection

**Table 2:** Photo protective activities of Mexican plants.

Plant	Photoprotective Activity	Photoprotective Agent	Biological Model Used	Reference
*Buddleja scordioides*	Reduction of skin damage, antioxidant activity, UVB absorption.	Methanolic extract Verbascoside, linarin, linarin acetate	*E. coli*, guinea pigs.	[83]
*Buddleja cordata*	Reduction of skin damage, antioxidant activity, UVB absorption.	Methanolic extract Verbascoside	SKH-1 hairless mice, mouse fibroblasts.	[84,85,86]
*Echinacea* spp.	Reduction of skin damage, antioxidant activity.	Methanolic extract, hydroxycinnamic acids, flavonoids	Clinical trials, in vitro antioxidant tests.	[90,91,92,93]
*Galinsoga parviflora* and *Galinsoga quadriradiata*	Skin photoprotection, antioxidant activity	Aqueous extract, caffeic acid derivatives.	NHDF human skin fibroblasts.	[102,103]
*Helianthus annuus*	UVB absorption, antioxidant activity, prevention of UVB damage and photoaging.	Flower ethanolic extract, chlorogenic and caffeic acid	Human dermal fibroblast, in vitro antioxidant and SPF tests.	[108]
*Larrea tridentata*	Inhibition of UVB induced signaling pathways related to skin carcinogenesis, antioxidant activity	Nordihydroguayaric acid (NDGA)	Human keratinocyte cell line HaCaT, in vitro antioxidant tests.	[113,114,115,116,117,118]
*Lippia graveloens*	UVB and UVC absorption, photochemopreventive and antioxidant activity.	Flavonoids, apigenin, pinocembrin and naringenin.	*E.coli*, SKH-1 mice, in vitro antioxidant assays.	[121,122]
*Malphigia glabra*	Antioxidant activity	Dried extract	in vitro antioxidant and SPF tests	[127]
*Pipper umbellatum*	Antioxidant activity, photoaging reduction, inhibition of metalloproteinases activity, reduction of skin cancer markers.	Ethanolic root extract, 4-nerolidylcatechol	Hairless mouse skin and SPF tests	[133,134,135,136,137]
*Polypodium leutocomos*	Skin antioxidant, antiphotoaging and cytoprotective activity.	Hydroxycinnamic acids, hydrophilic extract.	Human fibroblasts and keratinocytes	[146,147,148,155]
	Oral photoprotective effect: antioxidant, increment of p53+ cells, reduce proliferating cells. Reduction of systemic UVR damage.	Hydrophilic extract.	Hairless rats, clinical studies	[149,150,151,152,153,154]
*Portulaca oleacera*	Antioxidant, photoprotective, anti-wrinkle, anti-aging	Methanolic extracts	Epithelial keratinocytes and fibroblasts.	[160,164,165,166]
*Solanum lycopersicum*	Oral photoprotective and anticancer effect.	Tomato carotenoids enriched extract, lycopene.	Human volunteers, SKH-1 murine model.	[170,171,172]
	Skin photoprotection and antioxidant effect	Lycopene	Human dermal fibroblasts.	[173]
*Spondias purpurea*	UVA and UVB light protection, antioxidant effect	Methanolic extract, flavonoids, quercetin, rutin.	in vitro antioxidant and SPF tests	[176,178,180]
*Theobroma cacao*	Oral photoprotective effect. Anti-wrinkle effect, UVB radiation absorption, antioxidant capacity.	Cocoa beverage flavonols enriched. Cocoa pods extract	Human clinical trials in vitro antioxidant, antiwrinkle and SPF test	[48,183,184]
*Yucca periculosa*	Skin photoprotective effect, absorption of UVB radiation	Methanolic extract, polyphenols, resveratrol, methoxystilbene.	Guinea pigs	[186]
*Opuntia* spp.	Antioxidant properties, reduced lipid peroxidation and GSH depletion, inhibit UVB-induced skin degeneration	Aqueous extract, eucomic and piscidic acids, opuntiol, flavonols.	Human keratinocytes, HaCaT cells and hairless mice, NIH-3T3 cells	[192,193,194]

## 5. Conclusions

Exposure to UV radiation is a major risk factor for the development of skin cancer and related illnesses. Several compounds present in plants have been shown to have positive effects on skin health as well as photoprotective properties. It has been shown that several classes of secondary metabolites, such as alkaloids, carotenoids and phenolic compounds, provide protection against UVR damage in the skin. These compounds can absorb UVR, block it and stop the mechanisms by which UVR damages the skin.

Mexican plant biodiversity is a valuable source of secondary metabolites with photoprotective effects. Several Mexican plants produce unique molecules or mixtures with significant antioxidant and photoprotection properties. Due to their outstanding mechanisms of action, crude plant extracts, isolated compounds from plants or mixtures could all be potential ingredients in a new generation of sunscreens. In addition to their strong antioxidant and photoprotective properties, they also prevent photoaging and skin cancers. Furthermore, most of them have little to no side effects, making them a safe option for sunscreens.

There are undoubtedly many endemic plants in Mexico with photoprotective capabilities yet to be dissected. Additional research regarding the mechanisms of action, efficacy and dosage of photoprotective molecules and extracts of various Mexican plants is still required. Mexican plant biodiversity has the potential to be an outstanding source of UVR protectant molecules.

## Figures and Tables

**Table 1:** Ultraviolet radiation classification, characteristics and harmful effects ^1^.

Type of UVR	Characteristics	Acute Harmful Skin Effects	Chronic Harmful Skin Effects
Ultraviolet A radiation (UVA) 315 to 400 nm	Is not filtered by the stratospheric ozone layer in the atmosphere 90–99% reaches the earth’s surface Can penetrate deeper into the skin	Immediate pigment darkening Tanning	Photoaging: skin elasticity reduction and increase wrinkling. Immunosuppression
Ultraviolet B radiation (UVB) 280 to 315 nm	Filtered by the stratospheric ozone layer in the atmosphere 1–10% reaches the earth´s surface Can penetrate the upper layers of the epidermis	Edema, erythema, darkening, sunburns. Thickening of the epidermis and dermis.	Photoaging Immunosuppression Skin cancer
Ultraviolet C radiation (UVC) 100 to 280 nm	Completely filtered by the stratospheric ozone layer in the atmosphere Major artificial sources are germicidal lamps	Burn	Skin cancer

^1^ Modified from Narayanan et al. [1].

## Data Availability

Not applicable.
